# Innovative applications of silicon dioxide nanoparticles for targeted liver cancer treatment

**DOI:** 10.3389/fbioe.2025.1595772

**Published:** 2025-05-12

**Authors:** Tiantian Fu, Boshi Duan, Peng Sun, Wei Ma, Tianzuo Wang, Tianyou Wang, Zhuang Tong, Yue Wang

**Affiliations:** ^1^ Department of Thoracic Radiation Oncology Ward 1, Cancer Hospital of Dalian University of Technology, Liaoning Cancer Hospital & Institute, Shenyang, China; ^2^ Department of Medical Oncology, National Cancer Center/National Clinical Research Center for Cancer/Cancer Hospital & Shenzhen Hospital, Chinese Academy of Medical Sciences and Peking Union Medical College, Shenzhen, China; ^3^ Department of Hand Surgery 4 Ward, Central Hospital Affiliated to Shenyang Medical College, Shenyang, China; ^4^ Department of General Surgery, Cancer Hospital of Dalian University of Technology, Liaoning Cancer Hospital & Institute, Shenyang, China; ^5^ ShenYang No.126 Middle School, Shenyang, China; ^6^ Department of Thoracic Surgery, Cancer Hospital of Dalian University of Technology, Liaoning Cancer Hospital & Institute, Shenyang, China

**Keywords:** liver cancer, nanomaterial, treatment, silicon dioxide, nanosystem

## Abstract

Liver cancer remains a major global health challenge, characterized by high mortality and limited treatment efficacy. Conventional therapies, including chemotherapy, immunotherapy, and viral vectors, are hindered by systemic toxicity, drug resistance, and high costs. Silica nanoparticles (SiO_2_NPs) have emerged as promising platforms for liver cancer therapy, offering precise drug delivery, stimuli-responsive release, and integrated diagnostic-therapeutic capabilities. This review critically examines the potential of SiO_2_NPs to overcome these therapeutic limitations. Notable advances include their high drug-loading capacity, customizable surface modifications, and dual-responsive systems (pH/redox/NIR-II) that enable >90% tumor-specific drug release. Preclinical studies have demonstrated synergistic efficacy in combination therapies. Additionally, theranostic SiO_2_NPs enable MRI-guided tumor delineation and real-time treatment monitoring. Despite promising results, challenges remain in long-term biosafety, scalable synthesis, and regulatory compliance. Early-phase clinical trials, including those using NIR-II-responsive platforms, highlight their translational potential but underscore the need for further validation of toxicity profiles and manufacturing standards. Future research should focus on optimizing combinatory treatment strategies, scaling up production, and aligning with evolving regulatory frameworks. By bridging nanomaterial innovation with clinical applications, SiO_2_NPs offer unparalleled potential for advancing precision oncology in hepatocellular carcinoma.

## 1 Introduction

Liver cancer remains one of the most formidable global health challenges, ranking among the top ten most prevalent cancers and accounting for 9.1% of cancer-related deaths worldwide, approximately 830,000 deaths annually. The 5-year survival rate for liver cancer is less than 20% (Akinyemiju et al., 2017). The global incidence is approximately 906,000 new cases per year, with a corresponding death toll of 830,000 (Sung et al., 2021). Current therapeutic approaches predominantly include surgical resection, chemotherapy, radiotherapy, and interventional treatments, utilizing chemotherapeutic agents such as atezolizumab, nivolumab, pembrolizumab (Keytruda), and regorafenib (Stivarga) (Li et al., 2017). However, despite these efforts, high rates of postoperative recurrence continue to significantly impact patient prognosis and survival, highlighting the urgent need for novel and more effective therapeutic approaches (Llovet et al., 2005). Chemotherapy is often hindered by issues such as drug resistance and severe side effects. Clinical trials, including the Keynote-240 trial, have demonstrated substantial resistance and adverse reactions to agents like atezolizumab (Zhu et al., 2018). In contrast, the IMbrave150 trial reported that combination therapy with atezolizumab and bevacizumab significantly improved median overall survival to 19.2 months, compared with 13.4 months for sorafenib, providing a notable survival benefit (Cheng et al., 2022). These factors contribute to suboptimal treatment outcomes and diminished quality of life for patients.

**FIGURE 1:**
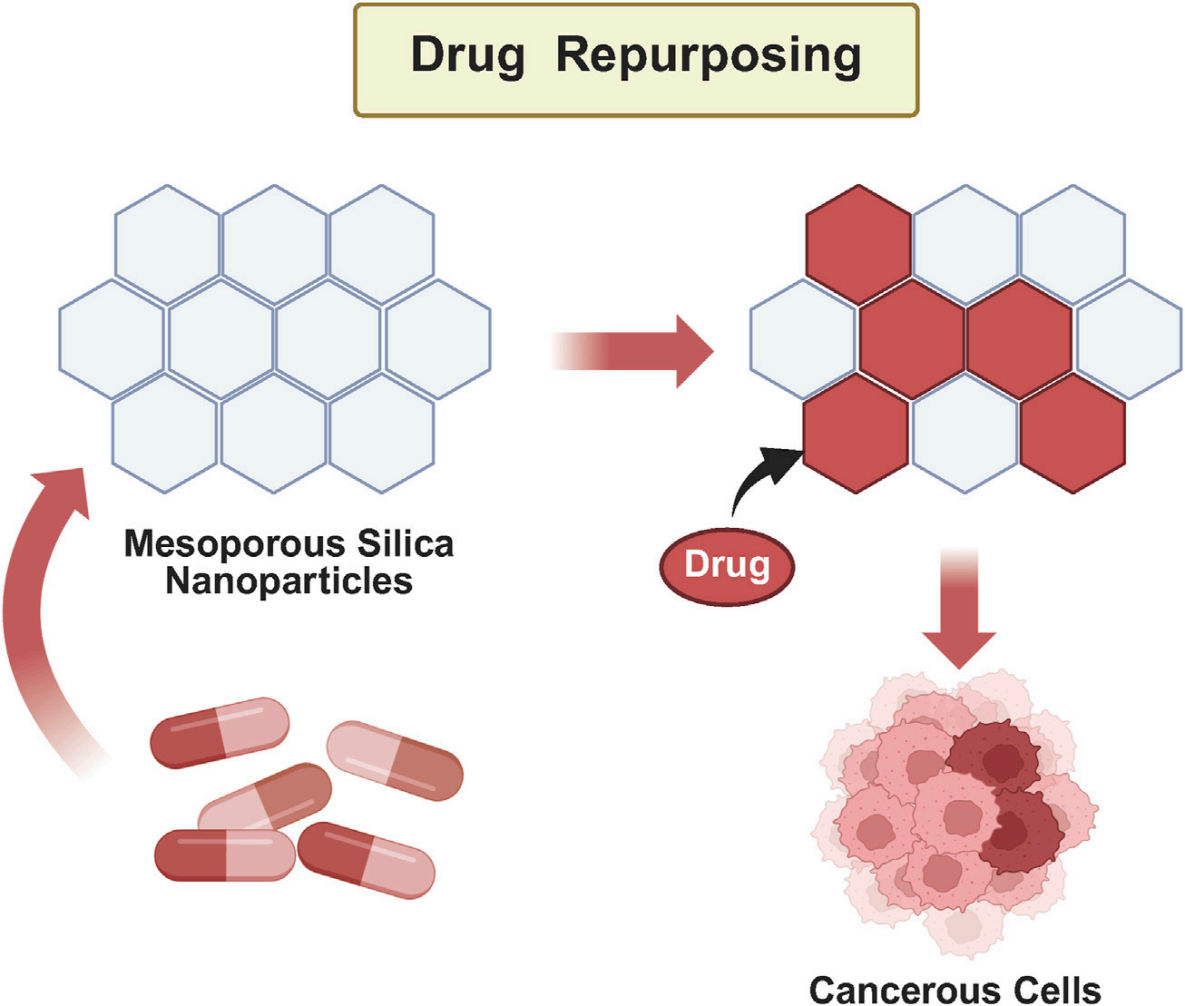
Graphical abstract.

Nanotechnology holds significant promise for advancing liver cancer treatment (Wang et al., 202a). Among the various nanoparticles under investigation, SiO_2_NPs have garnered considerable attention due to their unique physicochemical properties. These nanoparticles offer precisely tunable size and morphology, low cytotoxicity, exceptional biocompatibility, and stability under physiological conditions, making them well-suited for biomedical applications. Current research focuses on the potential of SiO_2_NPs in drug delivery systems (DDS), image-guided therapy, and photothermal treatment (Tarn et al., 2013; Anglin et al., 2008; Sadri et al., 2018). SiO_2_NPs can function as carriers for targeted drug delivery and controlled release, thereby improving therapeutic efficacy while minimizing adverse side effects. Additionally, their role in photothermal therapy enables precise tumor cell ablation through light-induced heat generation, a key feature in enhancing the specificity and effectiveness of cancer treatments. When compared to other nanoparticles, such as liposomes and gold nanoparticles, SiO_2_NPs demonstrate distinct advantages (Meng et al., 2022). For instance, SiO_2_NPs have shown superior drug-loading efficiency (25%) and better *in vivo* stability than liposomes (10%), making them more effective for sustained drug release and improved therapeutic outcomes (Portela et al., 2020). Their high contrast and biocompatibility also make them ideal for image-guided therapies, allowing for real-time monitoring and precise tracking of treatment progress. SiO_2_NPs represent a promising frontier in the fight against liver cancer (Li et al., 202a). In contrast to conventional therapeutic approaches, silica nanoparticles offer distinct advantages in drug delivery. Small molecule chemotherapy agents, such as doxorubicin, often face challenges such as rapid clearance and non-specific biodistribution (Zhong et al., 2021), while antibody-based therapies, such as nivolumab, are limited by high production costs and restricted tumor penetration. In contrast, SiO_2_NPs enable controlled drug release and dual-targeting capabilities via surface modification. However, to fully unlock their clinical potential, further research and rigorous clinical validation are essential. This review aims to comprehensively examine the role of SiO_2_NPs as drug delivery systems in liver cancer therapy (Figure 1), providing novel insights into their integration with diagnostic and therapeutic strategies to drive innovation and improve patient care.

## 2 Comparative analysis of hepatocellular carcinoma treatment approaches

### 2.1 Small molecule drugs

Small molecule chemotherapeutic agents, such as sorafenib, are currently the first-line treatment for hepatocellular carcinoma (HCC), but they have significant limitations. First, small molecules rely on passive diffusion to enter tumor tissues, lacking targeted delivery, which results in widespread distribution throughout the body and induces severe toxic reactions. For instance, the Phase III clinical trial of sorafenib (SHARP trial) revealed high incidences of adverse reactions, with dose reductions or discontinuations due to toxicity occurring (Llovet et al., 2008). Furthermore, the low drug loading and rapid metabolic clearance further limit their therapeutic efficacy, especially in advanced liver cancer, where high interstitial tumor pressure and dense stromal barriers significantly reduce drug penetration efficiency (Li et al., 2024b).

### 2.2 Antibody-based drugs

Antibody-based drugs, such as the PD-1 inhibitor nivolumab, achieve high targeting specificity through antigen-antibody binding, but their clinical application faces two major challenges. First, the production costs of monoclonal antibodies are extremely high, limiting their accessibility (Lee et al., 2023). Second, immune-related adverse events (irAEs) are frequent. In the Keynote-240 trial, the incidence of grade 3–4 irAEs (such as hepatitis and pneumonia) in the nivolumab treatment group was 18%, significantly higher than the 5% observed in the chemotherapy group (Finn et al., 2020). Additionally, the large molecular size of antibody drugs limits their tumor penetration, particularly in poorly vascularized or fibrotic liver cancers, further restricting their therapeutic efficacy (Debien et al., 2021).

### 2.3 Viral vectors

Viral vectors, such as adeno-associated virus (AAV), show potential in gene therapy, but their safety concerns must not be overlooked. AAV naturally infects hepatocytes, but the risk of random genomic integration may lead to insertional mutations. In a study using AAV2 for gene transfer to the liver, two participants experienced brief increases in liver enzyme levels, likely caused by immune rejection of the transduced hepatocytes, possibly mediated by AAV capsid-specific CD8^+^ T cells (Balwani et al., 2023). Furthermore, seven recombinant adeno-associated virus (rAAV)-based gene therapies have received regulatory approval; however, their clinical application remains constrained by safety concerns associated with high-dose viral administration. Notably, adverse effects such as hepatotoxicity, thrombotic microangiopathy (TMA), genotoxicity, and neurotoxicity have been documented. These issues underscore the need for careful consideration of immunogenic responses and the potential for serious side effects in patients undergoing treatment with these viral vectors (Wang et al., 2024b).

### 2.4 SiO_2_NPs

SiO_2_NPs offer significant advantages in liver cancer treatment due to their unique physicochemical properties (Liu et al., 2025). One key feature is their high drug loading capacity and controlled release properties. For example, a study demonstrated the use of PEG-modified arsenic trioxide (As_2_O_3_)-loaded mesoporous SiO_2_ nanoparticles for liver cancer therapy. This system exhibited excellent drug release characteristics in acidic environments, showcasing a high drug loading capacity and the ability to control drug release under pH-responsive conditions (Liu et al., 2025). Similarly, another research developed a pH/reduction dual-responsive mesoporous SiO_2_ nanoparticle platform for multi-drug co-delivery. This platform efficiently released drugs in both acidic and reductive environments, achieving up to 94% drug release in the tumor microenvironment (Yan et al., 2020). In addition to their therapeutic potential, SiO_2_NPs have applications in integrated diagnosis and therapy. For instance, Gd_2_O_3_-doped SiO_2_ nanoparticles were engineered to function as both magnetic resonance imaging (MRI) contrast agents and drug carriers. Although the specific drug loading capacity was not disclosed, the study highlighted their promising role in liver cancer imaging (Shao et al., 2011). SiO_2_NPs are known to accumulate in the liver, which may induce oxidative stress and fatty degeneration (Li et al., 2025). However, challenges such as batch-to-batch consistency in large-scale production and regulatory compliance remain important hurdles for the clinical translation of SiO_2_NP-based therapies (Rodríguez-Gómez et al., 2025).

### 2.5 Clinical significance of systematic comparison

In contrast to the systemic toxicity associated with small-molecule drugs and the cost constraints of antibody-based therapies, SiO_2_ nanoparticles present a promising approach to achieve a balance between therapeutic efficacy and safety through targeted delivery and controlled release mechanisms. For example, a study reported that PEGylated nanoparticles exhibited a significantly longer circulation half-life compared to uncoated nanoparticles. Specifically, uncoated nanoparticles had a circulation half-life of approximately 0.89 h, whereas PEGylated nanoparticles with mushroom and brush PEG configurations showed half-lives of 15.5 and 19.5 h, respectively (Suk et al., 2016). The synthesis of silica (SiO_2_) nanoparticles using microfluidic technology has been shown to achieve a low coefficient of variation (CV), contributing to batch-to-batch consistency in large-scale production. Future clinical trials are needed to further validate their clinical advantages. For instance, studies have demonstrated that SiO_2_ nanoparticles can serve as carriers for PD-1 inhibitors, enhancing their accumulation and efficacy in the tumor microenvironment (Liu et al., 2023).

## 3 The application of SiO_2_NPs in drug delivery systems

SiO_2_NPs consist of nanostructured materials composed of silicon and oxygen atoms, which combine in specific proportions to form stable structures. The unique properties and potential applications of these nanoparticles have attracted significant attention and research in scientific and engineering fields (Vivero-Escoto et al., 2010). Variants of SiO_2_ NPs, including nanoparticles, nanowires, and nanosheets, display diverse morphologies, depending on the methods used for their preparation.

### 3.1 Synthesis and performance regulation

SiO_2_ nanoparticles (SiO_2_NPs) have demonstrated significant potential in tumor diagnosis and therapy owing to their outstanding biocompatibility and tunable physicochemical properties (Fiorentis et al., 2023). The primary synthesis methods for SiO_2_NPs include sol-gel, microfluidic technology, and biomolecular templating. The sol-gel process entails the conversion of a silicon precursor, such as tetraethyl orthosilicate (TEOS), into nanoscale SiO_2_ particles through hydrolysis and condensation reactions. Both the TEOS concentration and the molar ratio of ammonia water catalyst have a substantial impact on particle size. For instance, one study utilized the sol-gel method to synthesize spherical SiO_2_ nanoparticles and investigated the effects of varying molar ratios of ammonia, TEOS, and ethanol on particle size (Guo et al., 2018). The results revealed that these molar ratios had a profound impact on both the size and morphology of the nanoparticles. Microfluidic technology, on the other hand, allows for precise control over fluid flow, enabling the production of SiO_2_NPs with a narrow size distribution. The SiO_2_ nanoparticles produced by this method were pseudospherical in shape, with an average particle size ranging from 10 to 150 nm. The particle size distribution was relatively uniform, with a specific surface area of approximately 360 m^2^/g (Luo et al., 2011). Moreover, the microfluidic device efficiently generated highly monodisperse spherical droplets, achieving a coefficient of variation (CV) of less than 2% for the step method and less than 5% for the flow-focusing nozzle. The droplets exhibited an average diameter ranging from 90 to 190 μm across both configurations (Shieh et al., 2021).

Additionally, tumor tissues typically exhibit an acidic microenvironment (pH∼6.5) due to increased glycolytic activity and hypoxia. pH-responsive SiO_2_NPs are engineered to release their therapeutic payloads in response to this acidic condition (Chu et al., 2022). The mechanism involves the protonation of ionizable groups on the nanoparticle surface, leading to structural changes that facilitate drug release. Studies have demonstrated that such pH-responsive SiO_2_NPs can effectively deliver anticancer drugs, resulting in enhanced tumor accumulation and therapeutic outcomes. The tumor microenvironment (TME), characterized by elevated levels of glutathione (GSH), hydrogen peroxide (H2O2), and an acidic pH, supports the growth, invasion, and metastasis of cancer cells (Zhang et al., 2022). GSH-responsive SiO_2_NPs utilize disulfide bonds that are cleaved in the presence of high GSH concentrations, triggering the release of encapsulated drugs (Zhou et al., 2022). This redox-sensitive mechanism ensures that drug release occurs predominantly within the tumor, where GSH levels are elevated, thereby reducing off-target effects and enhancing therapeutic efficacy. Finally, integrated diagnostic and therapeutic designs, by incorporating magnetic materials (e.g., Fe_3_O_4_) or fluorescent probes, endow SiO_2_NPs with multimodal imaging capabilities (Xie et al., 2024). Fe_3_O_4_-SiO_2_NPs demonstrate strong tumor recognition ability in MRI imaging. These surface modifications and performance enhancements significantly elevate the potential of SiO_2_NPs in targeted liver cancer therapy.

### 3.2 Targeted modification

Targeted modification of SiO_2_NPs involves the attachment of specific chemical groups or biomolecules to their surfaces, facilitating precise interactions with target substances or biological systems (Selvaggio et al., 2020). This approach enhances the directed application of the nanoparticles’ properties and functions. Surface modification of mesoporous SiO_2_NPs is essential for altering surface reactivity, improving biocompatibility, and extending *in vivo* circulation time (Schmidt et al., 2017). Ligand-targeted agents that bind to cancer cell receptors enable receptor-mediated cellular uptake, thereby enhancing targeted drug delivery and therapeutic efficacy. Various molecules, including peptides, small molecules, antibodies, and vitamins, can be conjugated onto nanoparticle surfaces (Tao et al., 2015).

Surface modification and performance optimization are pivotal strategies for enhancing the precision diagnosis and treatment of liver cancer using SiO_2_ nanoparticles (SiO_2_NPs) (Roh et al., 2023). One key modification, PEGylation, involves the attachment of polyethylene glycol (PEG) chains to nanoparticles, significantly improving their biocompatibility, prolonging their plasma half-life, and increasing tumor accumulation. This modification reduces immune responses and minimizes non-specific uptake by the mononuclear phagocyte system (MPS), thereby enhancing drug delivery efficiency. The first FDA-approved PEGylated nanoparticle formulation, Doxil^®^, was approved in 1995, and its “Stealth^®^” liposomes increased the bioavailability of doxorubicin nearly 90-fold within 1 week post-injection compared to the free drug. Furthermore, the drug demonstrated a half-life of 72 h, with a circulation half-life of 36 h. The extended circulation time enhances the probability of nanoparticle accumulation in tumor tissues through the enhanced permeability and retention (EPR) effect, where the leaky blood vessels in tumors preferentially permit the accumulation of larger particles (Greish, 2010). In addition to PEGylation, targeted ligand modification, such as conjugation with GPC3 antibodies or folate receptors, further enhances the selective uptake of SiO_2_NPs by liver cancer cells, improving their tumor-suppressive efficacy (Bakrania et al., 2021). For instance, multifunctional nanoparticles targeting GPC3, a proteoglycan associated with hepatocellular carcinoma (HCC), have shown enhanced cellular uptake in GPC3-positive HCC cells, highlighting their potential for targeted drug delivery and imaging applications (Liu et al., 2024). Moreover, Wu et al. (2015) synthesized mesoporous silica nanoparticles (MSNs) loaded with doxorubicin (DOX) and modified with PEG, integrating them with copper sulfide (CuS) nanoparticles to create efficient drug delivery platforms.

Folate has emerged as a prominent targeting ligand, owing to the overexpression of folate receptors (FR) on liver cancer cells (Ebrahimnejad et al., 2022). Folate receptors, particularly FR-α, are membrane-bound glycoproteins that play crucial roles in DNA synthesis, cell proliferation, DNA repair, and intracellular signaling—processes that are essential for tumorigenesis (Holm and Hansen, 2020). Notably, FR-α stimulates the proliferation of hepatocellular carcinoma (HCC) cells via the PI3K/Akt signaling pathway, positioning it as a promising and effective target for liver cancer therapy (Juliano, 2016). For example, Zhang et al. (2021a) developed nanoparticulate arsenic trioxide prodrugs (NiAsOx) encapsulated within large-pore mesoporous silica nanoparticles, with magnetic iron oxide nanoparticle surfaces (M-LPMSN-NiAsOx). The incorporation of folate onto these nanodrugs significantly enhanced their cytotoxic effects compared to free arsenic trioxide, resulting in increased cell apoptosis. *In vivo* studies using H22 tumor-bearing mice demonstrated the superior antitumor efficacy of M-LPMSN-NiAsOx-FA, while also offering exceptional capabilities for real-time tumor monitoring (Wang et al., 2014). In addition to folate-targeted strategies, Wu et al. (2015) developed PEGylated mesoporous silica nanoparticles (MSN) loaded with doxorubicin (DOX) and integrated with copper sulfide (CuS) nanoparticles (PEG-MSN/DOX/CuS). This system exhibited enhanced therapeutic efficacy through the synergistic combination of photothermal therapy and chemotherapy for hepatocellular carcinoma. However, a direct comparison between M-LPMSN-NiAsOx-FA and PEG-MSN/DOX/CuS in terms of receptor binding affinity and *in vivo* distribution is essential to fully evaluate the advantages of different modification strategies. Wang et al. (2014) introduced a novel liver-targeted therapy using lactobionic acid conjugated to active-targeting mesoporous silica nanoparticles. This formulation not only extended the circulation time but also facilitated efficient accumulation at hepatic tumor sites. Upon reaching the tumor’s reductive microenvironment, the incorporated platinum (IV) underwent reduction, enabling the rapid release of active platinum (II), thereby enhancing therapeutic efficacy. Xie et al. (2014) developed doxorubicin-loaded mesoporous silica nanoparticles functionalized with carboxyl groups (DOX–MSN/COOH) for targeted imaging and therapy of hepatocellular carcinoma. *In vivo* experiments revealed significant tumor growth inhibition and prolonged survival in mice, surpassing the efficacy of free doxorubicin (Ye et al., 2018). Histological analysis showed no significant adverse effects on normal liver and cardiac cells, emphasizing the safety profile of the DOX-MSN/COOH system. Combination therapy has become an increasingly popular strategy for enhancing therapeutic efficacy in cancer treatment. Silica nanoparticles (SLNs) modified with low-density lipoproteins (LDL) have been proposed as a targeted delivery platform (LDL-SLN) for selective delivery to LDL receptors, which are notably overexpressed in hepatocellular carcinoma (Ghosh et al., 2023). A novel drug delivery system (DDS) based on LDL-SLN was designed for the co-delivery of sorafenib (Sor) and doxorubicin (Dox), enabling synergistic chemotherapy for HCC (Zhu et al., 2023). Compared to monotherapies with either Sor or Dox, the LDL-SLN/Sor/Dox system demonstrated significantly enhanced antitumor efficacy in both *in vitro* and *in vivo* models.

### 3.3 Stimuli-responsive controlled release

Stimuli-responsive silica nanoparticles (SR-SiNPs) are emerging as promising agents in tumor therapy, offering targeted treatment through both intrinsic and extrinsic stimulus-responsive mechanisms (Thews and Riemann, 2019). The tumor microenvironment is characterized by factors such as low pH, hypoxia, elevated glutathione (GSH) levels, and increased hydrogen peroxide concentrations, all of which influence the behavior of these nanoparticles. Tumor tissues exhibit significant pH variations due to heightened metabolic activity, with extracellular pH fluctuating between 6.5 and 7.2 (Tang et al., 2023). Lysosomal pH is further reduced to 5.0–5.5. pH-responsive nanoparticles have been extensively utilized in tumor therapy, capitalizing on these pH differences (Bruschi, 2015). Upon entering tumor tissue, they undergo structural changes triggered by the acidic microenvironment, leading to controlled drug release or activation. This mechanism enhances drug concentration in tumor tissue, minimizing side effects on normal tissues and improving therapeutic efficacy. To quantitatively analyze drug release kinetics, mathematical models such as the Peppas equation are employed (Bayer, 2023). These models describe the release mechanisms based on the diffusion and swelling behavior of the nanoparticles, providing insights into the rate and extent of drug release under varying pH conditions. For example, studies have shown that curcumin release from nanoengineered polysaccharides best fits the Korsmeyer-Peppas equation, with diffusional exponents indicating a non-Fickian diffusion mechanism (Niu et al., 2021).

Tumor cells exhibit higher GSH concentrations due to increased metabolic activity, presenting an opportunity for redox-sensitive drug delivery systems (Mirhadi et al., 2020). Nanoparticles incorporating disulfide bonds or other redox-sensitive linkers can respond to elevated GSH levels by releasing the encapsulated drug (Zhao et al., 2015). However, a potential limitation is the risk of off-target effects, as normal tissues may also have elevated GSH levels. To mitigate this, dual-responsive systems that combine redox sensitivity with other stimuli, such as pH or light, have been developed. For example, Zhao et al. (2021) developed a dual-stimuli-responsive delivery system using mesoporous silica nanoparticles (MSN) functionalized with hyaluronic acid and disulfide linkers. This system showed restricted drug release under pH 7.4 and pH 5.0 conditions, but accelerated release in the presence of GSH, with further enhancement when both GSH and hyaluronidase were present. In CD44 receptor-overexpressing HCT-116 cells, the system exhibited improved uptake via CD44 receptor-mediated endocytosis compared to thiol-functionalized MSN. pH/GSH dual-responsive SiO_2_ NPs can reduce off-target release to 5% (Guo et al., 2021). In addition to endogenous stimuli, exogenous stimuli systems, such as NIR light-responsive nanoparticles, provide more precise control over drug release, further enhancing the effectiveness of targeted therapy. External stimuli such as light, temperature, magnetism, and ultrasound provide precise control over drug release from nanoparticles (Guo et al., 2021). Among these, near-infrared (NIR) light-responsive systems are particularly advantageous due to their ability to effectively penetrate tissues, enabling targeted drug release. Near-infrared (NIR) light, with a wavelength range of 780–1,700 nm, is regarded as the ideal therapeutic window for light-activated drug delivery systems *in vivo*, as it provides deep tissue penetration while minimizing cellular damage (Tang and Wang, 2021). Nonetheless, the penetration depth of NIR light remains limited: NIR-I (700–900 nm) can reach depths of 1–6 mm, whereas NIR-II (1,000–1,700 nm) allows penetration up to 20 mm, thereby enhancing both imaging and therapeutic potential (Chen et al., 2021).

### 3.4 Gene delivery

Gene therapy involves introducing specific DNA fragments, small interfering RNA (siRNA), or antisense oligonucleotides into cells to modulate gene expression or target genes associated with diseases (Abhange et al., 2021). Silica nanoparticles have emerged as promising vectors in this process, serving not only as drug carriers but also as effective vehicles for gene delivery, particularly in liver cancer treatment. Loading siRNA onto silica nanoparticles enhances its efficacy by facilitating entry into liver cancer cells and protecting it from nucleases (Xie et al., 2023). Compared to traditional viral vectors, silica nanoparticles offer significant advantages in gene therapy. They avoid potential side effects associated with recombinant viral vectors, such as mutagenicity and toxic immune reactions (Su et al., 2021). Additionally, the precise control over the structure and properties of silica nanoparticles enhances their stability and selectivity during gene delivery, providing a reliable and safe platform for gene therapy applications. Zhang et al. (2021b) utilized polyethyleneimine-modified mesoporous silica nanoparticles (PMSNs) as versatile nanocarriers capable of simultaneously delivering plasmid DNA and cisplatin. Administration of HNF4α-loaded PMSNs to CD133-expressing Huh7 cells resulted in significant effects: suppression of proliferation, depletion of cancer stem cell (CSC) populations, attenuation of stemness-associated gene expression, and enhancement of mature hepatocyte-associated gene expression (Tsai et al., 2019). Furthermore, co-delivery of HNF4α-encoding plasmids and cisplatin via PMSNs induced S-phase cell cycle arrest and apoptosis in Huh7 cells (Tsai et al., 2019). Notably, dual-loaded PMSNs demonstrated superior efficacy in restraining tumor progression *in vivo*. Hepatocyte nuclear factor 4 alpha (HNF4α) plays a crucial role in suppressing HCC development by inhibiting hepatocyte epithelial-mesenchymal transition (EMT) and CSC generation through the inhibition of β-catenin signaling pathways (Ning et al., 2010).

To improve liver cancer-specific delivery, the incorporation of targeting ligands, such as antibodies or peptides, that selectively bind to receptors overexpressed on liver cancer cells can enhance the selectivity and uptake of silica nanoparticles. For instance, functionalizing nanoparticles with ligands targeting CD133, a marker of liver CSCs, can enhance the delivery of therapeutic agents specifically to CSC populations, potentially improving treatment outcomes and reducing off-target effects (Liu and Qian, 2021). Xue et al. developed HMSNs coated with lipid and loaded with doxorubicin hydrochloride (DOX) and miR-375 (LHD/miR-375) to target multidrug-resistant HCC (Xue et al., 2017). LHD/miR-375 effectively delivered both agents into MDR HepG2/ADR cells or HCC tissues, enhancing DOX uptake and inhibiting HCC cell growth. Long-term toxicity evaluations revealed that the LHD/miR-375 treatment group exhibited an ALT level of 45 ± 5 U/L in mice, showing no significant difference compared to the control group. While the study reported no significant toxic effects in HCC xenografts or primary tumors after combined treatment, comprehensive long-term toxicity evaluations are essential. Future studies should include assessments of liver and kidney function markers, as well as immunogenicity evaluations, to ensure the safety profile of such nanocarriers (Ferguson and Waikar, 2012). Quantitative comparisons between SiO_2_NPs and lipid nanoparticles (LNPs) are crucial to evaluate their relative merits as non-viral gene delivery vectors. Head-to-head studies assessing transfection efficiency, such as siRNA silencing efficiency, and cytotoxicity, including determining the lethal dose for 50% of cells (LD50), provide valuable insights.

### 3.5 Integrated diagnosis and treatment

The integration of diagnostic and therapeutic modalities, known as theranostics, marks a significant advancement in personalized medicine, enabling the seamless combination of diagnostic imaging with targeted therapy for more precise and effective treatment strategies (Kelkar and Reineke, 2011). Nanoparticles play a pivotal role in this integration, serving as carriers that facilitate both precise imaging and effective treatment delivery (Li et al., 2018). Advanced imaging technologies—including optical, radioisotope, CT, MRI, and ultrasound imaging—provide comprehensive biological insights, aiding clinicians in accurately assessing patient conditions and localizing lesions. When combined with nanoparticles, these imaging modalities offer enhanced capabilities, particularly in real-time surgical guidance. Introducing nanoparticles into the body allows clinicians to monitor tumor localization and boundaries in real time, enabling precise tumor resection and improving procedural efficacy and safety (Srivastava et al., 2023). Zeng et al. developed arginine-glycine-aspartic acid (RGD)-conjugated mesoporous silica nanoparticles (MSNs) loaded with high concentrations of indocyanine green (ICG) (Zeng et al., 2016). In murine models, these nanoparticles facilitated precise delineation of liver cancer margins, enhancing the contrast between tumor and normal tissue during imaging (Srivastava et al., 2023). Andreou et al. (2016) developed surface-enhanced Raman scattering (SERS) nanoparticles as molecular imaging probes for liver malignancies using silica-encapsulated SERS nanoparticles. Upon intravenous administration, Raman imaging readily identified liver tumors in both animal models. To enhance the analytical performance of SERS-based immunoassays, incorporating materials like molybdenum disulfide (MoS_2_) has been shown to improve sensitivity, achieving detection limits comparable to or better than traditional methods such as ELISA (Terzapulo et al., 2024). For instance, a study demonstrated that integrating MoS_2_ into SERS immunosensors enhanced the limit of detection for alpha-fetoprotein (AFP), highlighting the potential of SERS in early cancer detection (Er et al., 2021). Hu et al. (2019) developed upconversion nanoparticle (UCNP) coated with caffeine and glypican-3 (GPC3) antibodies, serving as both photosensitizers and targeting agents. These nanoparticles facilitated targeted photodynamic therapy (PDT) upon exposure to 808 nm near-infrared (NIR) laser irradiation, offering enhanced tissue penetration. However, the effectiveness of NIR light penetration is limited by tissue depth. To overcome this limitation, combining PDT with other imaging modalities, such as MRI, has been proposed to enhance treatment efficacy. For example, several studies demonstrated that Gd^3+^-modified SiO_2_ NPs enabled MRI-guided PDT, resulting in improved tumor targeting and therapeutic outcomes (Zeng et al., 2022a; Zeng et al., 2020).

## 4 Challenges and future perspectives

### 4.1 Current challenges

Silica nanoparticles (SiNPs) have emerged as promising agents in liver cancer therapy due to their favorable properties, including biocompatibility and tunable physicochemical characteristics. However, their clinical application necessitates addressing several critical challenges. While SiNPs exhibit low acute toxicity, studies demonstrate persistent accumulation in organs such as the liver and spleen. Smaller particles induce dose-dependent hepatotoxicity through mechanisms involving Kupffer cell activation and mitochondrial dysfunction. Batch-to-batch inconsistencies in size and surface charge remain unresolved, directly impacting biodistribution profiles and therapeutic efficacy. The absence of standardized protocols and guidelines for nanocarrier development and testing can result in inefficiencies in research and development processes. Current guidelines lack nanoparticle-specific protocols for elemental impurity assessments, creating approval bottlenecks. The absence of standardized characterization methods can hinder the translation process and introduce inconsistencies in data, complicating the comparison of results across various studies. Addressing these challenges requires the establishment of standardized synthesis protocols, comprehensive long-term toxicity studies, and the development of nanoparticle-specific regulatory frameworks to facilitate the clinical translation of SiNP-based therapies.

### 4.2 Translational roadmap

To accelerate clinical translation, we propose a three-phase development framework aimed at bridging the gap from bench to bedside. In the preclinical phase (2024–2026), the focus will be on establishing ISO/TC229-compliant synthesis protocols using AI-optimized microfluidics. In the early clinical phase (2027–2030), we will implement adaptive trial designs with real-time pharmacokinetic (PK) and pharmacodynamic (PD) monitoring to ensure efficient progression through clinical stages. Finally, in the post-marketing phase (2031+), we aim to develop blockchain-based traceability systems to enhance safety surveillance and ensure ongoing monitoring of product performance in real-world settings.

### 4.3 Priority research directions

#### 4.3.1 Foundational research priorities

We recommend the following priority directions. First, establish multi-omics platforms integrating single-cell RNA sequencing (e.g., ×10 Genomics) and spatial metabolomics to map NP biodistribution at single-organelle resolution. Adopt the FDA’s Nanotechnology Risk Assessment Framework to develop organ-on-chip models simulating 5-year accumulation effects. These initiatives aim to enhance the precision and efficiency of NP design and evaluation, facilitating their safe and effective application in biomedical fields. Second, develop machine learning algorithms to predict optimal surface ligand combinations, reducing trial-and-error experimentation by 40%. Implement real-time Process Analytical Technology (PAT) sensors in microfluidic reactors to ensure <5% size variability. These strategies seek to streamline NP synthesis processes, improving reproducibility and scalability for clinical applications.

#### 4.3.2 Clinical translation imperatives

We propose the following strategic initiatives. Collaborate with the European Medicines Agency’s (EMA) Innovation Task Force to co-develop Quality by Design (QbD)-based regulatory pathways. These pathways should incorporate dynamic dissolution testing that mimics hepatic sinusoidal hemodynamics and conduct accelerated aging studies with nanoparticle-specific stability criteria. Additionally, propose a master protocol for clinical trials that includes adaptive dose escalation using Bayesian pharmacokinetic modeling and employs radiomics-based response monitoring, such as nanoparticle-enhanced MRI radiomics. These efforts aim to streamline regulatory processes and optimize clinical trial designs, thereby enhancing the efficacy and safety of NP-based therapies in clinical settings.

### 4.4 Cross-disciplinary convergence

We advocate for the formation of international consortia that address three critical interfaces in nanomaterials development and regulatory processes. First, the Materials-Biology Interface, which would involve establishing open-access databases cataloging structure-activity relationships, such as surface ζ-potential vs. hepatocyte uptake efficiency. Second, the Preclinical-Clinical Interface, aimed at creating a SiNP Safety Atlas that integrates preclinical data from over five species and early human trial results. Finally, the Regulatory-Industry Interface, proposing a joint FDA-EMA NanoAccelerator program that includes parallel scientific advice sessions and unified impurity testing standards (e.g., ICP-MS protocols for silica degradation products).

## 5 Clinical translation and future directions

### 5.1 Clinical translation

In liver cancer therapy, SiO_2_ nanoparticle-based drug delivery systems have shown considerable potential through a range of strategic approaches. Chen et al. developed the IDPC@Zr nanocomposite, which generates oxygen within the tumor microenvironment, alleviating hypoxia and enhancing therapeutic efficacy. In mouse models, the tumor suppression rate reached 92.14% (Chen et al., 2018). Zeng et al. (2022b) designed a dual-stimuli-responsive delivery system, utilizing the differences in pH and glutathione (GSH) levels within the tumor to achieve specific drug release in liver cancer cells, thereby significantly improving treatment outcome. Rastegari et al. (2021) employed polyethyleneimine-modified mesoporous silica nanoparticles to co-deliver plasmid DNA encoding HNF4α and cisplatin, resulting in significant inhibition of tumor proliferation in Huh7 cells. In the field of theranostics, Liu et al. developed mesoporous silica nanoparticles integrated with a gadolinium chelate and modified with RGD, enabling high-contrast MRI-guided tumor boundary tracing. In an orthotopic liver cancer model, the tumor-to-normal tissue signal ratio reached 5.3:1 (Anani et al., 2021).

The silica-based nanoparticle platform “Cornell Dots” has undergone Phase I clinical trials, including the study registered as NCT02106598. This trial assessed the application of ultrasmall core-shell fluorescent silica nanoparticles for image-guided sentinel lymph node biopsy in patients with head and neck melanoma (Zanoni et al., 2021). The results demonstrated that these nanoparticles enabled high-sensitivity sentinel lymph node visualization, including detection through intact skin, with no adverse events observed during the study. As more nanomedicines emerge, enhancing tumor specificity and reducing off-target toxicity will become crucial directions for clinical translation. Recent developments in near-infrared II (NIR-II)-responsive SiO_2_NPs are accelerating the transition from preclinical research to early-stage clinical trials (Chan and Chang, 2024). In the field of precision diagnostics, NIR-II-responsive SiO_2_NPs have shown promise in various applications. One study developed a nanotheranostic platform combining gold nanorods, silica, rhodamine B, and manganese dioxide, which exhibited tumor-specific responsiveness and efficient synergistic therapeutic performance. This platform highlighted the potential for multimodal imaging and synergistic chemodynamic/photothermal therapy, emphasizing the versatility of NIR-II-responsive SiO_2_ nanoparticles in both diagnostic and therapeutic applications (Wen et al., 2022). However, SiO_2_NPs still face numerous challenges in clinical applications, including the need for long-term toxicity risk assessments, complexities in fabrication and functionalization processes, regulatory compliance, and clinical translation. To further advance their use in liver cancer treatment, systematic toxicological studies must be conducted, standardized preparation and quality control processes established, and regulatory evaluation standards tailored to the specific characteristics of nanomedicines. Additionally, rigorously designed clinical trials are required to validate their safety and efficacy.

### 5.2 Future prospects

Currently, although large-scale public reports on the use of SiO_2_NPs in combination therapies and smart-responsive systems are limited, existing literature provides a theoretical foundation and experimental support for these strategies.

#### 5.2.1 Combination therapy: SiO_2_NPs carrying anti-angiogenic drugs and PD-1 inhibitors for synergistic treatment

Anti-angiogenic drugs inhibit tumor neovascularization, thereby restricting the supply of nutrients and tumor proliferation, while PD-1 inhibitors alleviate immune suppression of T cells, enhancing the tumor immune response. Guo et al. analyzed hepatocellular carcinoma patients treated with anti-PD-1 and lenvatinib, identifying a circulating CD8^+^ T cell population that plays a crucial role in treatment efficacy (Guo et al., 2025). However, to ensure a smooth transition from preclinical studies to Phase II clinical trials, regulatory agencies will need to provide clearer guidance on the safety evaluation and administration routes for nanoparticle carriers, and establish close collaboration mechanisms with pharmaceutical companies and research institutions.

#### 5.2.2 Smart-responsive systems: development of NIR-II responsive SiO_2_NPs for synergistic photothermal-chemotherapy of deep liver cancer

Recent studies have extensively explored chemodynamic agents based on various metal ion pairs (e.g., Mo^5+^/Mo^6+^, Mn^2+^/Mn^4+^, Fe^2+^/Fe^3+^, and Cu^+^/Cu^2+^) for tumor microenvironment modulation (Liu et al., 2019). This approach has demonstrated promising results in enhancing therapeutic efficacy. However, NIR-I light, with its limited tissue penetration depth and a maximum permissible exposure of 0.33 W cm^−2^, is less effective compared to NIR-II light (Zhang et al., 2021c). To address this challenge, Ma et al. (2019) synthesized copper sulfide (CuS) with NIR-II photothermal properties, functionalized with manganese dioxide nanoparticles, to facilitate glutathione (GSH) depletion and enhance CDT via NIR-II photothermal activation. In this system, Cu^2+^ is reduced to Cu^+^ by intratumoral GSH, which subsequently catalyzes the conversion of tumor-derived H_2_O_2_ into hydroxyl radicals for CDT. Huang et al. (2021) demonstrated that hyperthermia induced by CuS NPs can be utilized for photothermal therapy, simultaneously triggering the phase transition of materials for controlled drug release under 1,060-nm laser irradiation. In the acidity microenvironment, the CuS NPs released from NB/CuS@PCM NPs could degrade to Cu2+, then Cu2+ was reduced to Cu+ during the depletion of GSH. As Fenton-like catalyst, the copper ion could convert hydrogen peroxide into hydroxyl radicals for chemodynamic therapy. Moreover, the NB originated from NB/CuS@PCM NPs could increase the intracellular ROS content to improve the treatment outcome of chemodynamic therapy. The animal experimental results indicated that the NB/CuS@PCM NPs could accumulate at the tumor site and exhibit an excellent antitumor effect. Additionally, Zhang et al. (2021d). Developed a nanoplatform consisting of SiO_2_nanoparticles doped with copper sulfide (CuS) that are responsive to both pH and near-infrared II (NIR-II) light. Under acidic conditions (pH 5.0), the system demonstrated a high drug release rate, facilitating targeted chemotherapy. Additionally, upon exposure to NIR-II laser irradiation, the CuS component exhibited photothermal properties, enhancing the therapeutic effect. *In vivo* experiments confirmed the efficacy of this dual-responsive system in reducing tumor volume, while *ex vivo* tissue analysis indicated minimal off-target toxicity.

## 6 Conclusion

SiO_2_NPs are transforming liver cancer therapy by enabling precise drug delivery, controlled release, and integrated diagnostic and therapeutic applications. Their unique properties allow the development of highly targeted and effective treatment systems, enhancing existing therapies and paving the way for personalized nanomedicine solutions. Beyond academic research, SiO_2_ nanoparticles hold significant commercial potential, promising advancements in the pharmaceutical industry through the creation of stable and efficient drug carriers that improve treatment outcomes for liver cancer patients. Integrating SiO_2_NPs into clinical practice empowers healthcare professionals to design more precise and personalized treatment plans, enhancing patient satisfaction and quality of life while reducing side effects and treatment-related burdens. To fully realize their potential, future research must address challenges related to long-term safety, scalability, and regulatory compliance. Overcoming these obstacles will facilitate the seamless transition of SiO_2_ nanoparticle-based therapies from the laboratory to the clinic, ultimately revolutionizing the treatment landscape for liver cancer patients.
